# PRESENCE OF RISK FACTORS FOR SUICIDALITY IN RURAL-DWELLING OLDER ADULTS WITH HOARDING DISORDER

**DOI:** 10.1093/geroni/igae098.1838

**Published:** 2024-12-31

**Authors:** Branden Schaff, Ben Porter, Caitlyn Nix, Mary Dozier

**Affiliations:** MIssissippi State University, Starkville, Mississippi, United States; Mississippi State University, Mississippi State, Mississippi, United States; Mississippi State University, Mississippi State, Mississippi, United States; Mississippi State University, Mississippi State, Mississippi, United States

## Abstract

Because one in three older adults with hoarding disorder has co-morbid major depressive disorder, it is critical to understand the potential presence of additional risk factors for suicidal ideation in this population. There is currently a dearth of information about social cohesion risk factors for suicidality in older adults, particularly those living in a rural context. Rurality has the potential to exacerbate issues related to loneliness and access to instrumental support, both of which can increase risk for suicide. Forty-four older adults (mean age: 69, SD=9.43; 23% male; 20% Black or African American) with hoarding disorder completed a comprehensive baseline assessment as part of an in-home intervention study that included the Columbia-Suicide Severity Rating Scale, a semi-structured diagnostic interview, and the NIH Emotion Toolbox. No participants reported a history of suicide attempts, and only two participants reported a history of an aborted attempt; however, 30% reported having had the thought that they “wished to be dead or not alive” and 11% reported previously having had “active suicidal ideation with some intent to act.” Within our sample, 29% reported a previous depressive episode. On the NIH Emotion Toolbox, 33% were elevated on Loneliness, 33% were elevated on Perceived Rejection, 21% were low on Instrumental Support, and 33% were low on Social Satisfaction. A considerable percentage of participants reported social cohesion factors that could influence their risk for suicidal ideation. Targeted modular interventions aimed at mitigating loneliness and improving instrumental support may bolster the impact of evidence-based treatments within this population.

